# Schistosoma mansoni TGF-β Receptor II: Role in Host Ligand-Induced Regulation of a Schistosome Target Gene

**DOI:** 10.1371/journal.ppat.0020054

**Published:** 2006-06-16

**Authors:** Ahmed Osman, Edward G Niles, Sergio Verjovski-Almeida, Philip T LoVerde

**Affiliations:** 1 Department of Microbiology and Immunology, School of Medicine and Biomedical Sciences, State University of New York at Buffalo, Buffalo, New York, United States of America; 2 Departamento de Bioquímica, Instituto de Química, Universidade de São Paulo, São Paulo, Brazil; 3 Southwest Foundation for Biomedical Research, San Antonio, Texas, United States of America; University of Wisconsin-Madison, United States of America

## Abstract

Members of transforming growth factor-beta (TGF-β) superfamily play pivotal roles in development in multicellular organisms. We report the functional characterization of the Schistosoma mansoni type II receptor (SmTβRII). Mining of the S. mansoni expressed sequence tag (EST) database identified an EST clone that shows homology to the kinase domain of type II receptors from different species. The amplified EST sequence was used as a probe to isolate a cDNA clone spanning the entire coding region of a type II serine/threonine kinase receptor. The interaction of SmTβRII with SmTβRI was elucidated and shown to be dependent on TGF-β ligand binding. Furthermore, in the presence of human TGF-β1, SmTβRII was able to activate SmTβRI, which in turn activated SmSmad2 and promoted its interaction with SmSmad4, proving the transfer of the signal from the receptor complex to the Smad proteins. *Gynaecophoral canal protein (GCP),* whose expression in male worms is limited to the gynaecophoric canal, was identified as a potential TGF-β target gene in schistosomes. Knocking down the expression of SmTβRII using short interfering RNA molecules (siRNA) resulted in a concomitant reduction in the expression of GCP. These data provide evidence for the direct involvement of SmTβRII in mediating TGF-β–induced activation of the TGF-β target gene, *SmGCP,* within schistosome parasites. The results also provide additional evidence for a role for the TGF-β signaling pathway in male-induced female reproductive development.

## Synopsis

Schistosomes are multicellular parasites that infect 200 million people worldwide. Schistosome development in the human host likely involves host molecules that regulate biological processes of the parasite. Members of transforming growth factor-beta (TGF-β) superfamily play pivotal roles in development in multicellular organisms. TGF-β signaling requires ligand binding to a specific surface receptor, TGF-β type II receptor. The authors isolated the schistosome TGF-β type II receptor (SmTβRII), which was found to be biologically active and responded to stimulation by host TGF-β. The gynaecophoric canal is a ventral groove in the male worm in which the female must reside for sexual maturity. Gynaecophoral canal protein (GCP) is a protein whose expression in male worms is limited to the gynaecophoric canal and is implicated in female reproductive maturation. *GCP* expression was found to be regulated by human TGF-β. Knocking down the expression of SmTβRII resulted in a concomitant reduction in the expression of *GCP,* providing evidence for the direct involvement of SmTβRII-mediated, host TGF-β–induced regulation of schistosome gene expression. This study implicates the TGF-β signaling pathway in worm pairing, a prerequisite for female egg production. Because the eggs produced by the worm pairs are responsible for pathogenesis, the authors' research identifies potential targets for intervention.

## Introduction

Members of the transforming growth factor-beta (TGF-β) superfamily of secreted polypeptide growth factors play important and diverse roles in cellular growth, differentiation, extracellular matrix formation, and immunosuppression [1–8]. The classical paradigm of TGF-β signaling has been elucidated through numerous studies of different model organisms (for detailed reviews see [9–18]). TGF-β mitogenic effects are mediated through binding to the corresponding plasma membrane receptors of the serine/threonine receptor kinase family. This family of membrane receptors is divided into two subclasses of distinct but structurally related receptors. The type II receptor binds TGF-β and then recruits the corresponding type I receptor to form an active ligand–receptor complex, in which the type I receptor gets phosphorylated and activated by the type II receptor. Phosphorylation occurs on the serine and threonine residues in a domain in the juxtamembrane region called the GS domain [19,20]. The activated type I receptor relays the signal to the next component of the signaling pathway, a member of the Smad family of TGF-β signal transducers, the receptor-regulated Smad or R-Smad. Upon phosphorylation by the type I receptor, the R-Smad forms a hetero-oligomeric complex with a common Smad or a Co-Smad (Smad4) [21,22]. The newly formed Smad complex then translocates into the nucleus where it binds nuclear proteins that direct the Smad complexes to specific promoter sequence(s), where they regulate the transcription of the target gene(s) to exert the specific cellular responses triggered by ligand activation. Structurally, members of the receptor family consist of relatively short cysteine-rich, three-finger toxin fold extracellular domains [23], single transmembrane (TM) domains, and intracellular regions composed mainly of the serine/threonine kinase domains [24–26]. Not only the specificity of TGF-β signaling is determined by the type I receptor activation by type II receptor, but also the downstream transmission of signals by the type I receptor is dependent on its binding to and subsequent activation by type II receptor. In general, type II receptors are constitutively active kinases [19,27–29] that undergo reorientation of the intracellular domains upon ligand binding, which results in activation of the recruited type I receptor [30].

Flat worms of the genus *Schistosoma* cause schistosomiasis, a debilitating disease that afflicts 200 million people [31]. Growth, migration, and development of the parasitic worms in their host are believed to be controlled, in part, by signals received from the host. Schistosoma mansoni worms in their niche in the definitive host are bathed in host molecules (hormones, antibodies, cytokines, growth factors, etc.) Data to date indicate that schistosomes are in a dynamic process of receiving and responding to host molecules. Receptors present on the parasite surface process the repertoire of signals in such a way that promotes development, and guides the worms through their journey from site of infection to their final destination [32,33]. Furthermore, the absolute prerequisite for the female worm to reside within the gynaecophoric canal of the male worm, in order to develop and maintain its reproductive activity, highlights the significance of a set of self signals on the growth and development of the parasite and differentiation of its tissues [34].

The diverse effects produced by members of the TGF-β superfamily on a wide array of cell types stimulated the investigation of this signaling pathway as a plausible means of signal transmission that could be involved in the parasite growth and maturation. Earlier studies identified members of the TGF-β pathways in S. mansoni. A TGF-β type I receptor (SmRK1 or SmTβRI) was found to be localized to the parasite surface [35] and to mediate phosphorylation and subsequent activation of receptor-regulated schistosome Smad2 (SmSmad2) [36,37], but not SmSmad1 [36,38]. The molecular characterization of SmTβRI and the elucidation of its role in transmitting the signal in parasite tissues have encouraged the search for the type II receptor. One recent study reported the isolation of three transcripts encoding a TGF-β type II receptor: one, a full length receptor with 5′- and 3′-UTRs designated *SmRK2;* a longer form lacking the stop codon and a 3′-UTR *(SmRK2a);* and a third truncated variant *(SmRK2b)* that encodes the first 53 amino acid (aa) residues of the N-terminal extracellular domain (ECD), followed by an inserted ten-residue hydrophobic domain [39].

In addition to the upstream components such as the type II receptor, the search for TGF-β–responsive gene(s) is important since the modulation of target gene expression dictates the phenotypic outcome of growth factor activation based on the cellular context. To that end, searching protein databases for genes possibly regulated by TGF-β resulted in the identification of a protein homolog to a secreted matrix protein whose expression is greatly induced by TGF-β in human lung adenocarcinoma cell line (βig-h3) [40]. The schistosome counterpart is an 86-kDa glycoprotein that is expressed on the surface of adult female worms. In male worms, its surface expression is limited to the gynaecophoric canal (S. mansoni gynaecophoral canal protein [SmGCP]), the site of direct interaction between the mating pair [41]. SmGCP contains multiple short, conserved repeats that exhibit homology to the developmentally regulated neural cell adhesion molecule fasciclin-I from Drosophila melanogaster [42].

In this study, we report the isolation and characterization of the full-length coding sequence of S. mansoni TGF-β type II receptor (SmTβRII) that extends the cDNA sequence an extra 1.5 kilobases (kb) longer than was previously reported *(SmRK2a)*. The extra cDNA sequence encodes the correct C-terminal end of *SmRK2a,* a 3′-UTR, as well as the polyadenylation signal and poly A tail. We also demonstrate a role for host ligands in regulating schistosome gene expression by showing the direct involvement of SmTβRII in human TGF-β ligand binding. In addition, we report the identification of *SmGCP* [42] as a TGF-β target gene in the parasitic helminth *S. mansoni,* and we present evidence for its transcription activation by human TGF-β1 via an SmTβRII-dependent mechanism in the parasite itself.

## Results

### SmTβRII: Identification, Cloning, and Sequence Analyses of the cDNA and Description of Genomic Organization

Two overlapping expressed sequence tag (EST) clones (CD126244 and CD069595) showed similarity to part of the kinase domain of the TGF-β type II receptor from different organisms and were found to span a region in the intracellular kinase domain of the isolated receptor corresponding to base pairs (bp) 755–1,608 of the submitted cDNA sequence. The use of a PCR product derived from these EST clones as a screening probe for S. mansoni cDNA expression libraries resulted in the isolation of five positive clones. The isolated cDNA clones were sequenced from both strands, and the sequence data were analyzed using the National Center for Biotechnology Information “BLASTX” homology search of protein databases. It was found that these clones belong to TGF-β receptor family of type II subclass. Of these, a 3,932-bp cDNA clone was found to encode a protein of 815 aa, which contains the entire predicted coding sequence of TGF-β type II receptor. Sequence comparisons demonstrated that the schistosome receptor shows highest similarity to mammalian Activin type II receptors (rat, mouse, and human, respectively). These results were based on comparison of the kinase domains of the isolated clone and other members of type II receptor subfamily. However, the N-terminal ECD and the TM domains showed greatest similarity to Activin type II receptors from the spotted green puffer, *Tetraodon nigroviridis,* and the zebra fish, *Danio rerio,* and to BMP type II receptor of the giant pacific oyster, Crassostrea gigas. Further analysis using Dense Alignment Surface method (DAS; http://www.sbc.su.se/~miklos/DAS) [43] predicted a single TM domain spanning aa residues 139 to 164. The protein sequence apparently lacks membrane-anchoring motifs such as GPI (glycosylphosphatidylinositol)-anchoring signals or signal peptides.

**Figure 1:**
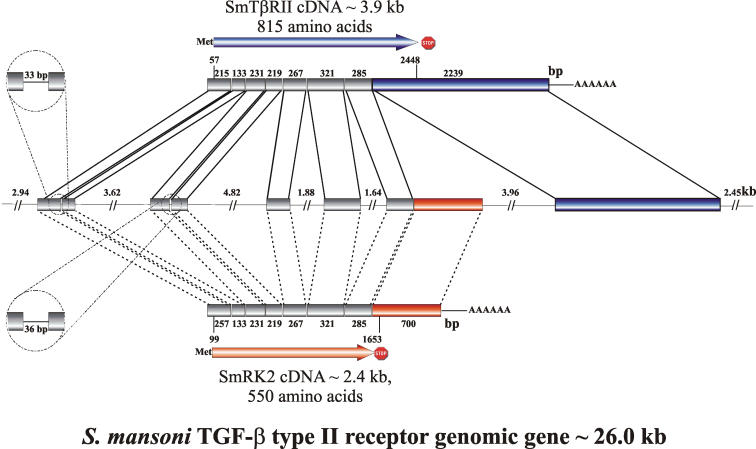
Genomic Structure of S. mansoni TGF-β Type II Receptor Gene A schematic representation of the genomic locus of S. mansoni TGF-β receptor II (middle) and the two alternatively spliced transcripts, *SmTβRII* (top) and *SmRK2* (bottom). The sizes of introns are represented in kb and that of exons (represented as cylinders) are in bp. The red exon is unique for *SmRK2* and the blue exon is unique for *SmTβRII*. Block arrows represent the two receptor isoforms, *SmRK2* (red) and *SmTβRII* (blue) with locations of start and stop codons indicated.

The cDNA clone reported in this study, SmTβRII, contains a 64-bp 5′-UTR and a 1.4 kb 3′-UTR, with a poly A tail and a putative “ATTAAA” polyadenylation signal located 21 bp upstream of the poly A tail. The use of the full-length SmTβRII cDNA sequence to screen S. mansoni genomic databases resulted in the assembly of several contigs to construct the genomic gene of TGF-β type II receptor (Figure 1). The 26 kbp gene consists of nine exons and contains about 2.9 and 2.4 kbp of genomic DNA located upstream and downstream of the first and last exon of SmTβRII, respectively. Analysis of the genomic sequence of SmTβRII and comparison to previously identified cDNA clones revealed the presence of two transcripts predicted to produce two different isoforms of type II receptor. One transcript encodes the sequence of the previously reported receptor (550 aa; *SmRK2*) [39]. A second, longer mRNA translates into the receptor described in this report (SmTβRII). These transcripts are produced from the same gene by alternative splicing of the last two exons (exons 8 and 9) (Figure 1). *SmRK2* and *SmRK2a* were reported to contain 100- and 105-bp 5′-UTRs, respectively [39]. Both cDNAs contain an extra 42 bp upstream of what was found in SmTβRII, which is also present in the genomic DNA and likely represents part of the first exon. The rest of the 5′-UTR of *SmRK2, SmRK2a,* and *SmTβRII* is almost identical (below).

The 5′-end of *SmRK2a* shows a 1-bp deletion at position 110, as compared to that of *SmRK2* and *SmTβRII,* which results in a frame shift that deletes amino acids 2 and 3, glutamate and cysteine. The sequence of the corresponding genomic DNA was found to match that of *SmRK2* and *SmTβRII,* but not *SmRK2a*. Therefore, we believe that the deletion could be attributed to a sequencing error of the *SmRK2a* cDNA. Regarding the 3′-ends, *SmRK2a* encodes a 754–aa protein that was proposed to lack the stop codon [39]. The authors explained that this transcript could represent a nonfunctional, rapidly degraded mRNA, or might encode a functional protein albeit lacking the stop codon [39]. DNA sequence data retrieved from genomic DNA databases revealed that the C-termini of *SmTβRII* and *SmRK2a* and the 3′-UTR of *SmTβRII* are encoded by exon 9 of the gene. Thus, these data indicate that the cDNA sequence of *SmRK2a* is incomplete and that *SmTβRII* sequence presents the missing 3′-end of *SmRK2a.*

The genomic sequence of the exon representing the region spanning the stop codon and the 3′-UTR reported in *SmRK2* [39], shows that what was thought to be a poly A tail in *SmRK2* is in fact a stretch of eight adenosine (A) nucleotides followed by two guanosines (G) then another three As (Figure S1A, pound [#] sign) and is not the actual poly A tail of *SmRK2* transcript. Further evidence for this is that a consensus polyadenylation signal could not be found in an appropriate location respective to the proposed poly A tail in *SmRK2* cDNA. It is likely that the approach employed to isolate the full-length cDNA, 3′ RACE (rapid amplification of cDNA ends) using poly dT primer, generated this error. Careful screening of the genomic sequence in search of putative polyadenylation signals for this transcript revealed the presence of a potential signal 309 bp downstream of the reported *SmRK2* poly A stretch (Figure S1A, asterisk [*]). In order to determine if this consensus sequence serves as an actual polyadenylation signal, we designed PCR primers upstream and downstream of it (Figure S1A; Table S1). When using cDNA templates, the expected PCR products could be amplified only when a forward primer (F1) was used in combination with either one of two reverse primers located upstream of the putative polyadenylation signal (R1 and R2; Figure S1B, panel I), but not with reverse primers located about 110 bp (R3) and 730 bp (R4) downstream of the proposed signal (Figure S1B, panels I and II). Similarly, no products were obtained when another forward primer (F2; a complementary sequence of R2) was used with reverse primers R3 and R4 (Figure S1B, panel III).

In addition, other PCR reactions were designed to address the relationship of the two transcripts. Primers F1, which is common for both *SmRK2* and *SmTβRII* (exon 7), and F2, which is unique for *SmRK2* and located close to the 3′-end of exon 8, were used with a reverse primer (R5), which is unique for SmTβRII (exon 9). Only F1×R5 (Figure S1B, panel II), but not F2×R5, reaction (Figure S1B, panel III), gave the expected PCR product with cDNA templates. These results indicate that the two transcripts are independently represented in the cDNA. Genomic DNA templates, either extracted genomic DNA or isolated bacterial artificial chromosome (BAC) clones, gave the expected products in all the reactions (Figure S1B). All PCR products were sequenced, and sequence information matched that obtained from the database.

The third truncated transcript reported in the Forrester et al. study [39] was described as encoding a 63 aa–long peptide and was suggested to function as a membrane anchor, act as a soluble ligand trap analogous to Follistatin, Noggin, or Decorin, or act as a membrane-bound accessory protein to promote ligand binding. This transcript, indeed, represents the first exon that covers the 5′-UTR and the following sequence that encodes the first 53 aa followed by a 33 bp–long intron that translates into 10 aa followed by a stop codon (Figure 1). The amplification of this DNA fragment could simply be attributed to contamination of cDNA preparations with genomic DNA that was carried over from total RNA extraction steps. This is consistent with the authors' notion that this sequence may originate from an unspliced intron, since it starts and ends with the classical GT-AG intron boundary sequences [39].

### Detection of Native SmTβRII Protein in Schistosomes

**Figure 2:**
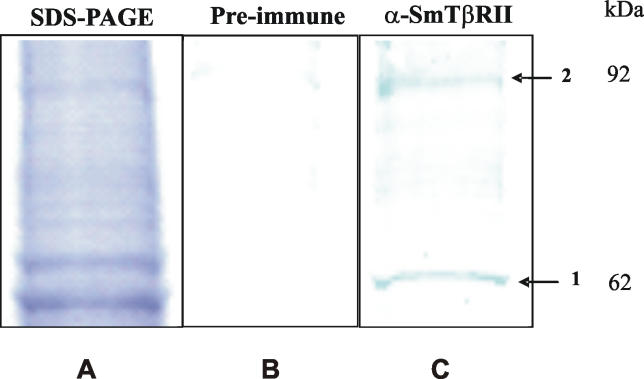
Identification of Native TGF-β Receptor II in NP-40 Extract of S. mansoni Adult Worm Pairs Coomassie blue–stained SDS gel (panel A), and Western blot analysis of NP-40 schistosome extract using an IgG fraction of pre-immune (panel B) and SmTβRII-immunized (panel C) rabbit sera. Arrows 1 and 2 point to 62 and 92 kDa isoforms, respectively.

SmTβRII was detected in schistosome extracts using affinity-purified IgG directed against the N-terminal 153 aa of SmTβRII, SmTβRII-N (41–193). In principle, the antibody used in these assays can detect both forms of type II receptor. As expected, two major protein bands were observed in Western blots using specific anti-type II receptor antiserum. The faster migrating component is about 62 kDa (Figure 2, panel C, arrow 1), whereas the higher molecular weight protein is about 92 kDa (Figure 2, panel C, arrow 2). These data provide further evidence for the presence of two related proteins. The shorter is 550 aa long (SmRK2; 62.2 kDa deduced size) described by Forrester et al., [39], and the larger is 815 aa (SmTβRII; 92.6 kDa deduced size). The detection of the two forms of the receptor in NP-40 extracts indicates that these receptors are associated with the tegument (outer covering) of the parasites. NP-40 extracts probed with pre-immune rabbit IgG, as a negative control, did not show any significant reactivity (Figure 2, panel B).

**Figure 3:**
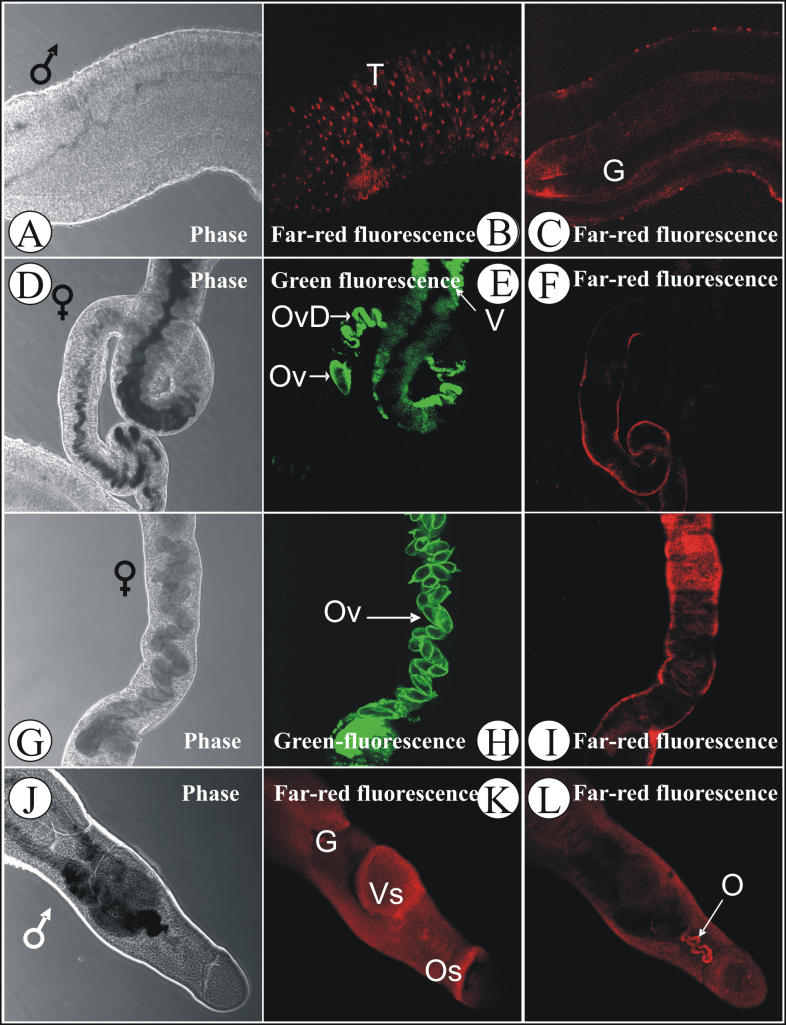
Whole-Mount S. mansoni Adult Worm Immunofluorescence S. mansoni adult worms were probed with anti-SmTβRII rabbit IgG and pre-immune rabbit IgG (Figure S2), followed by biotin-conjugated anti-rabbit IgG. Reactive complexes were detected using streptavidin Alexa Fluor 647 conjugate and analyzed with a Bio-Rad MRC1024 confocal laser microscope. Anti-SmTβRII reactivity is shown in a live male worm (♂) (panels A–C) in different laser sections in tubercles (T) (panel B) and gynaecophoric canal (G) (panel C). Specific surface fluorescence is also shown in live female worms (♀) (panels F and I), whereas green fluorescence fields show the non-specific auto-fluorescence in vitellaria (V), oviduct (OvD) (panel E), and ova (Ov) (panels E and H). An acetone-fixed male worm (♂) shows anti-SmTβRII reactivity in the gynaecophoric canal, oral (Os) and ventral suckers (Vs) (panel K), and in esophagus (O) (panel L). Panels A, D, G, and J are phase-contrast fields of the fluorescent fields B and C, E and F, H and I, and K and L, respectively.

In immunofluorescence assays, SmTβRII and/or SmRK2 could be detected in adult worm pairs in either cryosections (unpublished data) or in live or acetone-fixed whole parasites (Figure 3). Anti-receptor II antibody reactivity with native antigen(s) in live parasites was confined to the parasite surface in female worms (panels F and I) and in male worms, to the tubercles (panel B), as well as in the gynaecophoric canal (panel C). Antibody reactivity patterns in acetone-fixed parasites showed that receptor II is also localized at the interface between the parasite and its host, the oral and ventral suckers (panel K), and the lining tegument of the esophagus (panel L). No specific (far-red) fluorescence could be visualized when pre-immune rabbit IgG was used (Figure S2, panel C).

### Detection of SmTβRII Transcripts

**Figure 4:**
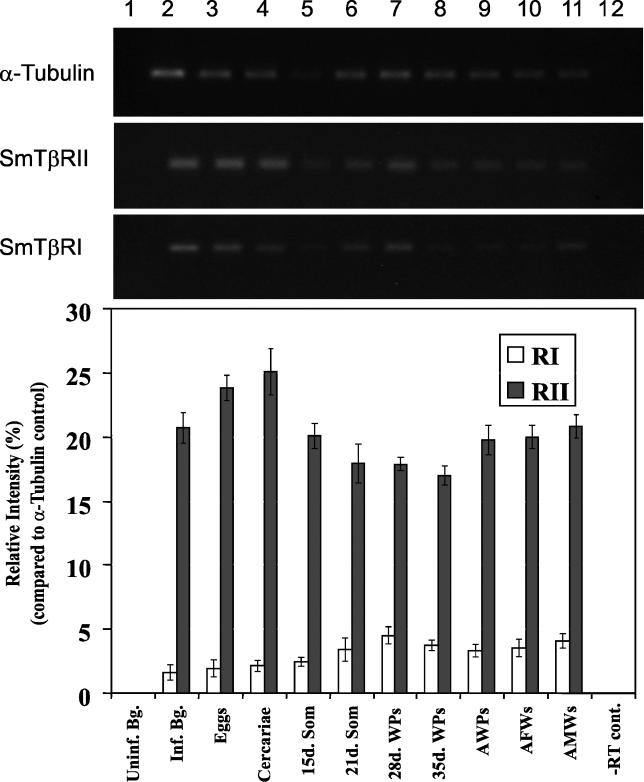
Semi-Quantitative RT-PCR Analysis of SmTβRII Top panel shows agarose gel separation of the PCR products of SmTβRII (middle), SmTβRI (bottom) and the constitutively transcribed control, α-tubulin (top). Lanes are numbered and the respective stages are listed at the bottom of the bar graph. The bar graph representation shows the percentage values of the optical densities in pixels of the PCR bands of SmTβRII (gray bars) and SmTβRI (white bars) compared to the corresponding band of α-tubulin control from the same stage. Values were calculated from three independent PCR amplifications (Error bars represent standard deviation values). Samples included in the assay were the hepato-pancreas regions of the intermediate host; uninfected and 30-d–infected B. glabrata snails, which represent different stages of daughter sporocysts; parasite eggs obtained from the liver of Syrian golden hamsters infected with 5,000 cercariae; cercariae shed and collected from infected snails; and stages representing different time points during schistosome development in mammalian host, obtained by perfusion of infected hamsters for the specified time (15–45-d-old worms). Adult male and female worms, separated after perfusion, were also included in the assay. Samples were subjected to total RNA extraction and cDNA synthesis, followed by PCR amplification using specific primer pairs.

A RT-PCR semi-quantitative analysis was employed to assess the level of receptor II mRNA throughout schistosome development. Compared to the constitutively expressed control, α-tubulin, *SmTβRII* exhibited relatively steady expression levels throughout development. On the other hand, *SmTβRI* showed lower expression levels in developmental stages at earlier than 15-d postinfection, while the overall expression pattern of *SmTβRI* was significantly lower than that displayed by *SmTβRII* (Figure 4).

**Figure 5:**
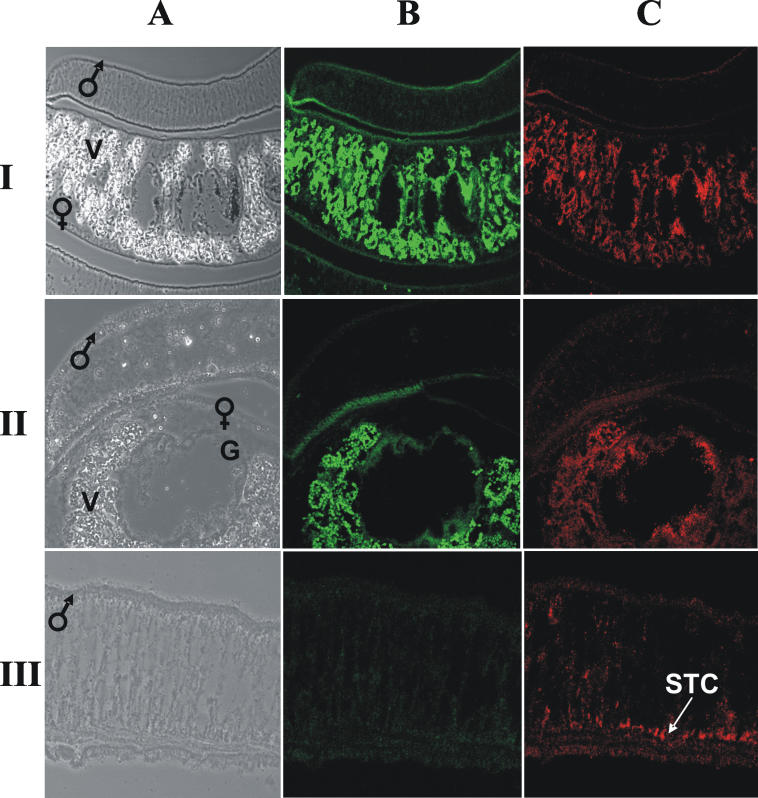
Localization of SmTβRII mRNA Transcripts in Tissue Sections of S. mansoni Adult Worms by FISH Column A represent phase-contrast fields, Column B represent non-specific autofluorescence fields observed in vitelline lobules (V) using green fluorescence filter (522 nm) and Column C shows specific probe reactivity as represented by far-red fluorescence using 680 nm filter. Row (I) shows sections of a male (♂) and a female (♀) worm probed with the positive control cRNA probe (the antisense strand of eggshell protein P14). Specific fluorescence could be observed in the vitellaria (V) of the female worm. As expected, no specific fluorescence could be observed in male worm sections. Specific reactivity of SmTβRII antisense probe could be seen in vitelline cells (V) and gut epithelial cells (G) in a female worm section (panel IIC) and in subtegumental cells (STC) in a male worm section (panel IIIC). No significant fluorescence could be seen in the negative control reaction using SmTβRII sense cRNA strand (unpublished data).

TGF-β type II receptor transcripts were also detected in adult worm pair paraffin sections. By using an anti-sense cRNA probe designed to the N-terminal region of *SmTβRII,* we detected the transcripts in the vitelline cells and gut epithelial cells (Figure 5, panel IIC), as well as subtegumental cells (Figure 5, panel IIIC). As expected, P14, an eggshell precursor transcript employed as a positive control, was localized to the vitelline cells in adult female worm sections with no reactivity in male sections (Figure 5, panel IC). No specific reactivity could be observed in the negative control sections probed with sense cRNA probe of SmTβRII-N (unpublished data).

### In Vitro Interaction of SmTβRII with SmTβRI

**Figure 6:**
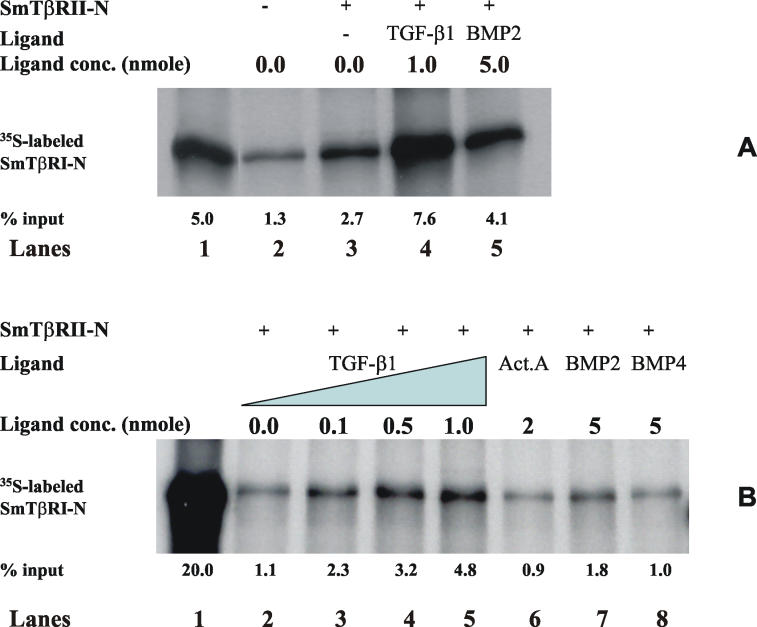
In Vitro Interaction of Amino- (N-) Terminal Domains of SmTβRI and SmTβRII Interaction of the in vitro translated non-labeled N- terminal domain of SmTβRII with ^35^S-labeled N-terminal domain of SmTβRI, in the presence or absence of different TGF-β ligands (panels A and B) or varied amounts of different TGF-β_1_ (panel B). Ligand concentrations (in nM) are shown at the top of each lane. Reactions were precipitated using S-protein agarose beads (EMD Biosciences, Novagen). Precipitated products were separated by SDS-PAGE and subjected to autofluorography. In vitro translated products (5% and 20 %) of input radiolabeled products are shown in the left lane of panels A and B, respectively. Background precipitation of ^35^S-labeled-SmTβRI-N by S-protein agarose beads is shown in lane 2, panel A. The background reactivity was removed in later experiments by pre-clearing ^35^S-SmTβRI-N by treatment with the S-protein agarose beads (panel B) prior to use in interaction assay. Percentage values of precipitated reactive radiolabeled product of each reaction are shown at the bottom of each lane.

Schistosome TGF-β receptor interactions in vitro were evaluated by co-immunoprecipitation. The degree of receptor interactions were expressed as the percentage values of the precipitated radiolabeled product compared to the input material. Incubation of the N-terminal domains of the two receptors resulted in a 2-fold enhancement (Figure 6, panel A, lane 3) over background precipitation of ^35^S-SmTβRI-N with S-protein agarose beads (Figure 6, panel A, lane 2). Non-specific background precipitation was removed in later experiments (Figure 6, panel B) by pre-absorbing ^35^S-labeled SmTβRI-N with S-protein agarose beads. Compared to the interactions in the absence of ligand, adding 1 nM of recombinant human TGF-β1 (Figure 6, panel A, lane 4 and panel B, lane 5) or, to a lesser extent, 5 nM recombinant human Bone Morphogenetic Protein-2 (BMP2; Figure 6, panel A, lane 5, and panel B, lane 7) significantly enhanced the interaction of type I and type II receptor N-termini. Furthermore, the receptor interaction showed a dose-dependent enhancement in response to TGF-β1 (Figure 6, panel B, lanes 2–5). Neither Activin A (2.0 nM) nor BMP4 (5.0 nM) stimulated N-terminal domain interactions (Figure 6, panel B, lanes 6 and 8, respectively). Thus, these results demonstrate that the interaction of the N-terminal regions of the two receptors is dependent on the presence of TGF-β ligand. Further interaction assays showed that the kinase domains of both receptors readily associate, while no significant interaction was observed between the receptors' C-termini (unpublished data).

**Figure 7:**
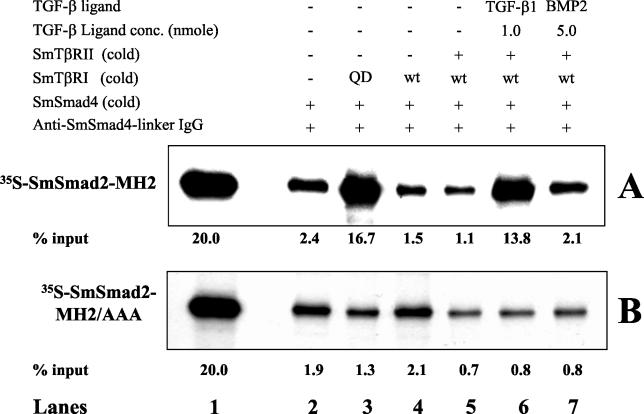
The Transduction of TGF-β Signal to SmSmad2 via Activated SmTβRII/SmTβRI Receptor Complex, In Vitro: Co-immunoprecipitation of SmSmad2-MH2 and SmSmad4 ^35^S-labeled, in vitro translated products of SmSmad2-MH2 (panel A) and SmSmad2-MH2/AAA (panel B) were incubated with SmSmad4 in the presence of SmTβR-I (wt) and SmTβR-II in the presence or absence of TGF-β1 (1.0 nM) or BMP2 (5.0 nM). Radiolabeled, in vitro translated products were co-precipitated with SmSmad4, using anti-SmSmad4-linker IgG and Protein A Sepharose beads (Amersham Biosciences). Background precipitation was removed by treating ^35^S-labeled in vitro translated products with anti-SmSmad4-linker IgG and Protein A Sepharose beads. The pre-cleared lysates were then used in the above-described reactions. A positive control reaction (lane 3) was included, in which SmSmad2-MH2, or the AAA mutant peptide, were reacted with SmSmad4 in the presence of the active mutant form of type I receptor, SmTβR-I (Q-D). Reactions, which contain either SmSmad2-MH2 or its AAA mutant form with SmSmad4 in the presence of wild-type SmTβRI, represent the negative controls of the assay (lane 4). Immunoprecipitated products were separated by SDS-PAGE and subjected to autofluorography. Lanes are labeled to specify the input components of each reaction. In vitro translated products (20% of input) are shown (lane 1). Percentage values of precipitated reactive radiolabeled product of each reaction are shown at the bottom of each lane.

These data demonstrate that human TGF-β1, and to a lesser extent human BMP2, could stimulate the interaction of the N-terminal domains of SmTβRII and SmTβRI, which suggests that the parasites may utilize the human growth factors in their developmental processes. The extent to which the signal could be transduced in the parasite tissues, which is whether or not TGF-β–induced receptor interaction is functional, was next investigated. In order to address this point, an SmSmad2/SmSmad4 interaction assay, similar to that previously described [38], was designed. In the previous report, the constitutively active, ligand-independent mutant form of type I receptor (SmTβRI-Q/D), but not wild-type SmTβRI (SmTβRI-wt) could stimulate the SmSmad2/SmSmad4 interaction. In this study, we adopted the same approach and examined the SmSmad2-MH2 interaction with SmSmad4 in the presence of wild-type receptors, SmTβRII and SmTβRI, in the absence or presence of either 1 nM human TGF-β1 or 5 nM human BMP2. Constitutively active and wild-type SmTβRI were used as positive and negative controls, respectively. In case of reactions containing both wild-type receptors in the absence of ligand (Figure 7, panel A, lane 5) or in the presence of BMP2 (Figure 7, panel A, lane 7), an SmSmad2/SmSmad4 interaction was obtained that is below that of the background reaction (in absence of receptors; Figure 7A, lane 2) or of the negative control reaction (in the presence of wild-type receptor I alone; Figure 7A, lane 4). In contrast, in the presence of TGF-β1, a significant increase in interaction of SmSmad2-MH2 domain with SmSmad4 was observed (Figure 7, panel A, lane 6), and was comparable to what was obtained in the positive control reaction with SmTβRI-Q/D (Figure 7, panel A, lane 3). On the other hand, when a mutant, non-phosphorylatable form of SmSmad2-MH2 domain (SmSmad2-MH2/AAA) was used, no significant enhancement in the interaction with SmSmad4 could be detected in any of the reactions containing type I receptor alone or along with type II receptor (Figure 7, panel B). Therefore, these data demonstrate that the TGF-β stimulated a functional interaction of type II and type I receptors, which in turn relayed the signal down to SmSmad2 and promoted its interaction with SmSmad4.

### Identification of *SmGCP* as a S. mansoni TGF-β Target Gene

**Figure 8:**
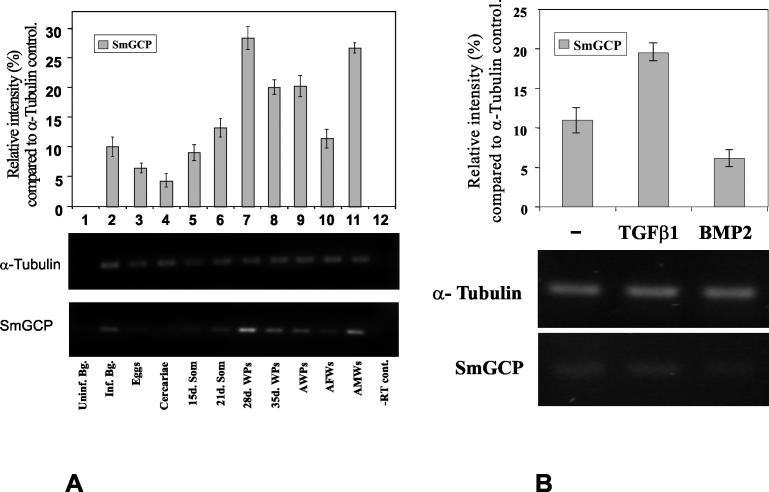
Semi-Quantitative RT-PCR Analysis of SmGCP mRNA The bottom panel shows the agarose gel separation of the PCR products of SmGCP (bottom), and the constitutively transcribed control, α-tubulin (top). Panel A: Lanes are numbered and the respective stages are listed at the bottom of the panel. Panel B: Adult worm pairs (42-d-old) were left untreated (lane 1) or treated with human TGF-β1 (1 nM; lane 2) or human BMP2 (5 nM; lane 3). Top of each panel shows a bar graph representation of the relative PCR band intensities (%) of SmGCP compared to that of α-tubulin control. Values were calculated from three independent PCR amplifications (Error bars represent the standard deviation).

SmGCP was found to exhibit homology to three related proteins: D. melanogaster fasciclin I [44]; mouse and OSF2 (human osteoblast specific factor 2) [45], a homophilic adhesion molecule that plays a role in bone formation; and lastly the βig-h3 [40]. Each contains repeated, short, highly conserved regions within more divergent homologous domains (fas1 domain) of approximately 140–150 aa [40,44]. SmGCP contains two of the fas1 domains between residues 43–306 and is localized to the adult parasite surface (restricted to the gynaecophoric canal in male worms), although it lacks a consensus TM domain or a GPI-anchoring signal [42]. The first step in evaluating the *SmGCP* gene as a possible target for TGF-β regulation was to determine its expression pattern throughout development. Semi-quantitative RT-PCR was performed in which *SmGCP*-specific primers were used to amplify a PCR product of approximately 300 bp. Figure 8A shows that *SmGCP* exhibits an expression peak at 28 d postinfection (lane 7), which coincides with worm mating. A similar expression level was observed in adult male worms (lane 11), although relatively low levels are observed in 15-d-old or earlier schistosomules and parasite eggs (lanes 3–5).

The responsiveness of *SmGCP* to induction by members of the TGF-β family was also examined. Total RNA was extracted from adult worms that were incubated in culture media supplemented with either 1 nM of TGF-β1 or 5 nM of BMP2, and subjected to semi-quantitative RT-PCR. Transcript levels for *SmGCP* and identified members of TGF-β signaling pathways were examined using this approach. Compared to untreated worm controls (Figure 8B, lane 1), an approximately 2-fold increase of *SmGCP* expression was observed in adult worms treated with human TGF-β1 (Figure 8B, lane 2), although BMP2 treatment resulted in a slight decrease in SmGCP levels (Figure 8B, lane 3). No significant differences were detected in levels of SmTβRI, SmSmad1, SmSmad2, or SmSmad4 compared to untreated controls (unpublished data).

The above data indicate that *SmGCP* expression is modulated by human TGF-β. To determine whether this is dependent on stimulation of the TGF-β signaling pathway or a secondary event incited through stimulation of a different signaling network as part of a more generalized cellular response, we investigated the effect of blocking TGF-β signaling on the expression of *SmGCP,* by employing RNA interference (RNAi) to knock down type II receptor as the initial event of TGF-β signaling.

**Figure 9:**
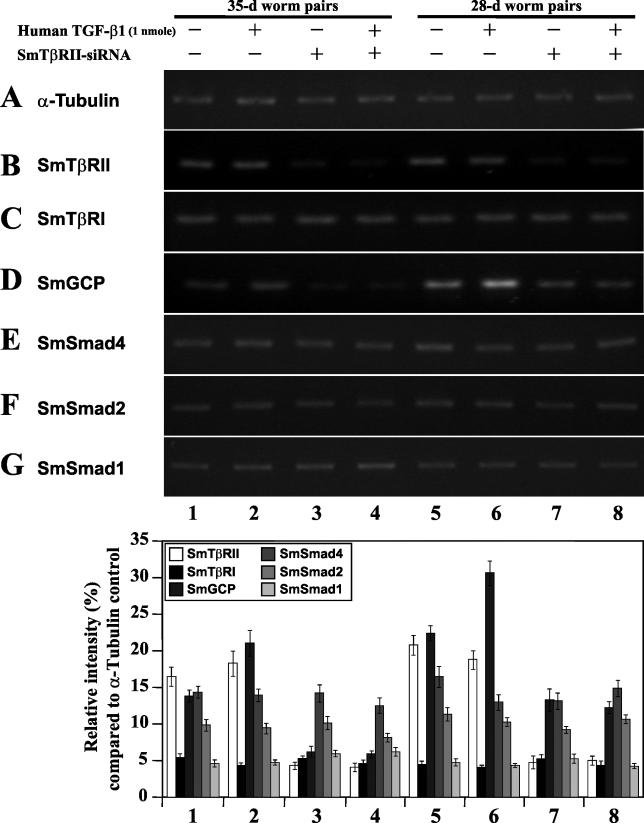
Silencing of TGF-β–Induced Expression of SmGCP by Knocking Down SmTβRII Expression Semi-quantitative RT-PCR analyses for transcripts of SmGCP as well as various components of schistosomal TGF-β signaling pathways in 35-d-old and 28-d-old old worm pairs, untransformed and transformed with SmTβRII-siRNA, and either left untreated or treated with TGF-β1 (1 nM). The top panel shows the agarose gel separation of the PCR products of SmTβRII (panel B), SmTβRI (panel C), SmGCP (panel D), SmSmad4 (panel E), SmSmad2 (panel F), SmSmad1 (panel G), and the constitutively transcribed control, α-tubulin (panel A). The lanes are labeled to show detailed treatment of each sample. The bar graph representation shows the percentage values of the optical densities in pixels of the PCR bands for each gene compared to the corresponding band of α-tubulin control from the same stage. Values were calculated from three independent PCR amplifications (Error bars represent the standard deviation).

Freshly perfused 26-d-old and 33-d-old worm pairs transformed with TGF-β type II receptor–specific short interfering RNA (siRNA) were left untreated or treated for 24 h with 1 nM human TGF-β1. RT-PCR data demonstrated that SmTβRII-specific siRNA treatment did diminish the levels of SmTβRII mRNA about 3- to 4-fold (25%–30% of the levels of untreated samples; Figure 9, panel B, lanes 3, 4, 7, and 8). Moreover, not only did SmTβRII-siRNA treatment result in a concomitant reduction in SmGCP levels (there was 2- to 3-fold reduction in levels of SmGCP compared to untreated samples; Figure 9, panel D, lanes 3 and 7), but SmGCP also failed to respond to human TGF-β1 induction (Figure 9, panel D, lanes 4 and 8) as opposed to what was observed in non-transformed parasites (Figure 8B, lane 2, and Figure 9, panel D, lanes 2 and 6). None of the other cDNA species that were used as controls for PCR amplification (SmTβRI, SmSmad1, SmSmad2, and SmSmad4) showed any significant variation that could be attributed to different treatment regimens (Figure 9, panels C, E, F, and G), indicating that the responses were specific and triggered by SmTβRII-siRNA treatment. These gene products, although part of the same pathway as SmTβRII, are not directly regulated by TGF-β, as mentioned above.

## Discussion

In this study, we report the identification of type II TGF-β receptor in *S. mansoni,* which represents a crucial step in elucidation of the source and subtype of the ligand(s) that regulate the biological events stimulated by these pathways and subsequently determine the ultimate outcome in terms of phenotype. Genomic DNA sequence information gathered from S. mansoni databases and validated by sequencing SmTβRII-positive genomic clones isolated from the BAC library revealed the genomic organization of the SmTβRII cDNA cloning and gene. RT-PCR data demonstrated the presence of two independent transcripts that give rise to two different TGF-β type II receptor isoforms. These transcripts are products of the same gene generated by alternative splicing of the last two exons. The amino acid sequence and BLAST search data show that SmRK2 [39] encodes a receptor that lacks the last 40 aa of the putative kinase domain. Consequently, the S. mansoni TGF-β type II receptor undergoes alternative splicing to produce transcripts that give rise to different length receptors that might exhibit different kinase activities. These different type II receptors may signal in different cells or developmental stages cooperating with SmTβRI or another unidentified type I receptor. Previous studies on Xenopus laevis activin receptor II, ActRII [46], and mouse ActRII gene [47], as well as human TGF-β type I receptor SKR2 [48], reported multiple mRNA variants that give rise to C-terminally truncated isoforms for each receptor. This led Xu and his coworkers [48] to propose that the addition of the poly A tail to alternative exons at the carboxyl-terminal coding exon–intron junction could represent a common feature of both TGF-β type I and type II receptor genes. Therefore, alternative splicing generates transcripts encoding products that vary in length and subsequently in their kinase activities and target phosphorylation sites, a feature that may add to the heterogeneity of biological effects of individual ligands.

The semi-quantitative RT-PCR results show that the type II receptor exhibits a relatively constant transcription level throughout the parasite lifecycle that is 3- to 5-fold higher than that displayed by SmTβRI. This suggests the presence of a second type I receptor that may cooperate with SmTβRII in the stages in which SmTβRI shows a significantly lower expression level, such as the early stages of development [35], and stages involving the intermediate host. Furthermore, SmTβRI was found not to mediate the signals transmitted through SmSmad1 [36,38], a fact that supports the presence of a second SmTβRI receptor capable of interacting with and activating SmSmad1. An understanding of the performance of the different type II receptor isoforms (SmRK2 and SmTβRII) in different signaling contexts and their cellular localization and expression patterns are issues that await further investigation. The phenomenon of alternative splicing, of different exons at the C-terminal end, was reported before in S. mansoni SER, an epidermal growth factor receptor ortholog [49].

The detection of both TGF-β type II receptor isoforms in the NP-40 extracts of adult worm pairs, which represents a pool of outer tegument proteins, provides evidence that both isoforms are in fact expressed in the adult stage. They are present in the tegument where the type II receptor, as the initial signaling component in the TGF-β pathway, is anchored into the surface and may respond to both host and self signals. Such expression profiles and the localization of TGF-β type II receptor at the interface between the parasite and its host was further confirmed by immunofluorescence assays on whole-mount adult worms as well as on adult worm sections. The detection of either or both of the two isoforms on the surface of live parasites (tubercles and gynaecophoric canal in male worms and general surface localization in females) and in the lining of the esophageal canal of fixed parasites (Figure 3), highlights the fact that the TGF-β type II receptor is actively engaged in dynamic signaling processes at the host–parasite interface. Our results are in agreement with localization data reported for SmRK2 that showed intense surface staining in both female and male worm sections, which indicated that SmRK2 exhibits tegumental expression [39]. It can be concluded from the report of Forrester et al. [39] that the observed expression pattern could be representative for either or both isoforms of type II receptor since the utilized antibody reagents used in that study were generated against an N-terminal synthetic peptide that is shared in both isoforms.

The fluorescent in situ hybridization (FISH) assay (Figure 5) provided an additional method for detection and localization of type II receptor transcript. The detection of the transcripts in the subtegumental cells is consistent with the production of SmTβRII in the tegument and its insertion into the parasite's surface. In addition, the type II receptor transcripts were also detected in gut epithelia and vitelline cells. This may indicate that the receptor could be involved in other signaling processes leading to sexual and digestive tissue development and differentiation. The type II transcript and protein distribution profiles are consistent with previous studies that reported the localization of components of TGF-β signaling pathway, SmTβRI [50], SmSmad2 [37], and SmSmad4 [38], to digestive tract and male and female reproductive tissues. Furthermore, the RT-PCR results demonstrate that type II receptor shows little variation throughout the parasite lifecycle, an observation that may indicate its involvement in parasite growth and development in different stages.

Interaction assays were designed to identify the domain(s) involved in receptor complex formation. Our results demonstrate that in the absence of ligand, full-length SmTβRI and SmTβRII showed no significant interaction. However, the ECD of the two receptors exhibited ligand-dependent interaction. Furthermore, the kinase domains of the receptors displayed autonomous interaction, whereas the C-termini showed no affinity toward each other. These data are in agreement with previous studies, which reported that the interaction between the TGF-β receptors depends on the ligand binding to either type II receptor followed by the recruitment of receptor I, as in the case with TGF-β and activin [19,51,52] or to both receptors as in the case of BMPs [53–55]. Several reports have also shown the interaction of the kinase domains of receptor I and receptor II for members of the TGF-β superfamily [56–58].

Furthermore, our results demonstrate that the presence of the appropriate ligand (TGF-β1 and to a lesser extent BMP2) stimulated the association of the ECD of receptor I and receptor II, whereas Activin A or BMP4 had no affect. These results were surprising since the BLAST search data categorize the S. mansoni type II receptor as belonging to the Activin type II receptor subfamily. A similar situation was found in *Drosophila* type II receptor, punt, which was initially classified as belonging to the Activin type II receptor subfamily, based on sequence similarities and ligand-binding properties [59]. However, this receptor was later found to bind to and mediate signals of the decapentaplegic (Dpp), a *Drosophila* ortholog of mammalian BMP, and functionally respond to members of the BMP family [53]. Thus, determination of the functional ligand can not be predicted based only on sequence similarities of the kinase domain. Moreover, Beall and Pearce [36] reported that human TβRII appears to facilitate TGF-β binding to SmTβRI and that a chimeric receptor composed of the extracellular domain of SmTβRI and the intracellular domain of human TβRI could bind human TGF-β ligand and activate a luciferase reporter system in response to TGF-β1 and TGF-β3 but not BMP7.

In our previous work, we reported that SmSmad2 associates with SmSmad4 upon phosphorylation and activation of the former by type I receptor, SmTβRI. Only the constitutively active mutant form of SmTβRI (SmTβRI/Q-D), but not the wild-type receptor, could achieve this activation in the absence of ligand or receptor II [38]. In this study we included the type II receptor and the ligand to evaluate Smad complex formation as a secondary event following receptor complex formation and receptor I activation of R-Smad. Our data show that TGF-β1 induced SmSmad complex formation comparable to what was observed with SmTβRI/Q-D, whereas in absence of ligand or in the presence of BMP2, SmSmad2/SmSmad4 association was at background level (in absence of receptors). These data demonstrate that host TGF-β1 not only induced receptor complex formation, but also propagated the signal to R-Smad causing its activation and association with Smad4. This is consistent with the results of Lagna et al. [21] who reported that agonist-induced activation of human Smad1 (by BMP) and Smad2 (by TGF-β and activin) led to their association with Smad4. On the other hand, the lack of interaction between the non-phosphorylatable mutant of SmSmad2 (SmSmad2-MH2/AAA) with SmSmad4 is in agreement with a previous report that showed that in the presence of BMP, mutation of the C-terminal phosphorylation motif of Smad1 abolished transduction by preventing its association with Smad4 [60].

The SmGCP exhibits surface expression in adult parasites especially in male worms in which expression is limited to the gynaecophoric canal and is almost absent in unisexual male worms [42]. Interestingly, the transcription profile of SmGCP shows its peak expression in 28-d-old worms, a time that coincides with worm pairing. This distribution pattern emphasizes the potential role of SmGCP in male–female interactions and the male-stimulated reproductive maturation of the female schistosome [34]. TGF-β1–treated worms displayed an enhanced expression level of SmGCP. In order to investigate the mechanism of SmGCP expression induction in response to TGF-β, we knocked down SmTβRII expression to prevent initiation of the TGF-β signaling pathway. We employed RNAi to achieve silencing of the SmTβRII gene, as the successful use of this technique to knock down gene transcription in S. mansoni sporocysts had been previously demonstrated [61,62]. In this study, we report the effective use of siRNA as a tool to knock down a target gene expression in late parasitic stages (28- and 35-d-old worms). The chosen delivery method, the particle bombardment, proved effective, as was shown in different studies reporting the use of biolistic technique to transfect S. mansoni with different constructs [63–68] and in our standardization experiments using an HcRed-encoding plasmid. The observed concomitant reduction of SmGCP and SmTβRII expression in worms treated with SmTβRII siRNA suggests that SmGCP closely follows the expression pattern of TGF-β receptor II. In fact, the failure of TGF-β1 to induce the expression of SmGCP in worms in which the type II receptor was knocked down indicates a direct relationship between the classical TGF-β pathway as represented by type II receptor and SmGCP as a potential target gene. However, SmGCP could represent a primary TGF-β target gene or it could be a target gene for another gene product that primarily induced by TGF-β pathway. A detailed investigation involving the assessment of schistosome Smad complex binding to the promoter region of SmGCP will be necessary to address this point at the molecular level. In either case, it is obvious that induction of SmGCP expression constitutes part of the mitogenic effect exerted by the host TGF-β ligand in schistosome.

In schistosomes, an interesting biological interplay has evolved such that male schistosomes, via an unknown stimulus, regulate female-specific gene expression and thus female reproductive development and egg production [34]. In order for the male to stimulate and to maintain female reproductive development, there must be direct contact between the male and the female. This is accomplished by the female residing in the gynecophoric canal of the male. One scenario is that the female first stimulates the male, and the male in turn produces signals that regulate female development [34]. The coinciding of peak expression of SmGCP with worm pairing, localization of SmGCP in male worms only in the gynecophoric canal [41] and the requirement for the male to maintain contact with the female, and induction of SmGCP by TGFβ implicates the TGFβ pathway as an important signaling component for worm pairing.

The induction of SmGCP expression by TGF-β1 provides support for the hypothesis that schistosome gene expression can be regulated by host ligands. It further implicates the host ligand as a stimulator for parasite–parasite interactions via regulating the expression of a gene that is likely involved in such biological events. Several studies have demonstrated involvement of host molecules in regulating biological processes in schistosomes. Host tumor necrosis factor-α (TNF-α), secreted as a part of the host response to eggs trapped in liver tissues, was found to significantly stimulate egg laying by adult female worms [69]. In addition, human EGF induced phosphorylation of native SER protein, the S. mansoni homolog of EGFR. In response to human EGF stimulation, SER was also shown to be able to activate a Ras-responsive reporter system in epithelial canine cells and mediate EGF responses in *Xenopus* oocytes [70]. The effects of thyroid hormone, interleukin-7, and insulin on schistosome development in the mouse host have also been demonstrated [71].

The demonstration that schistosome type II receptor is able to bind human TGF-β ligand provides strong evidence for the utilization of host ligand in parasite growth and development. However, this, in fact, does not rule out the involvement of a self ligand or a set of ligands, as localization studies for components of schistosome TGF-β signaling pathways indicate that TGF-β signaling is active in sexual tissues, a fact that implies active engagement of TGF-β signaling in internal parasite organs as well as at the interface with its host. This observation has been reported previously in a study that showed type II receptors have diverse ligand-binding abilities, and this phenomenon offers an explanation for the wide variety of biological responses that can be elicited by members of TGF-β family [53].

## Materials and Methods

### Identification, cloning, and sequence analyses of SmTβRII.

Mining of S. mansoni DNA sequence databases resulted in the identification of two overlapping EST clones (CD126244 and CD069595) that show similarity to different members of TGF-β type II receptors [72]. Based on the EST sequences, a primer pair was designed that amplified an 859-bp PCR product from adult worm cDNA, which was cloned in pCR2.1-TOPO vector (Invitrogen, Carlsbad, California, United States) and sequenced. The DNA fragment was labeled using the MegaPrime DNA labeling system (Amersham Biosciences, Piscataway, New Jersey, United States) and employed to screen an S. mansoni adult worm pair λ-ZapII cDNA expression library [73]. Isolated clones were excised in vivo and converted to pBlueScript SK+ phagmids, using ExAssist lambda helper phage following the vendor's instructions (Stratagene, Mountain View, California, United States). One of those clones was found to contain the entire coding sequence of the type II TGF-β receptor. DNA and protein sequence analyses were performed using programs in the Wisconsin Package Version 10.3 (Accelrys, San Diego, California, United States).

DNA sequence analysis revealed the presence of two SphI cut sites, one preceding the start ATG codon, while the second is about 525 bp downstream of the start ATG codon (at bp 59 and 592 of the submitted cDNA sequence). Two amplification primers were designed to amplify the cDNA fragment corresponding to the N-terminal domain of the receptor (193 aa); a forward primer that represented DNA sequence from bp 65–87 and had a BamHI cut site inserted upstream of the start ATG codon and a reverse primer, which represented the sequence complementary to bp 625–645 of the submitted cDNA sequence. The PCR product was cloned into the pCRII-TOPO vector (Invitrogen) giving rise to pSmTβRII-N(1-193)/TOPO-II that was sequenced to confirm the absence of PCR-generated errors, digested with BamHI and SphI, and re-cloned into the parental BlueScript-SK+ vector that was digested with the same enzymes to generate a modified version of the cDNA clone in which the 5′-UTR upstream of the start ATG codon was removed and a BamHI site was brought forward upstream and in frame with the start ATG codon. The modified vector, pSmTβRII-BlueScript-SK+, was digested with BamHI and with XhoI, which is located at the 3′-end of the multiple cloning sites of the parent vector pBlueScript-SK+, to excise the SmTβRII cDNA fragment suitable for cloning in several vectors designed with BamHI and either XhoI or SalI at the 5′- and the 3′-ends of the multiple cloning sites, respectively.

### Genomic organization of SmTβRII gene.

S. mansoni genomic DNA databases from The Wellcome Trust Sanger Institute and The Institute for Genomic Research (TIGR), were searched using the type II receptor cDNA sequence to identify contigs that constituted the entire gene. In addition, full-length cDNA was excised from the parent vector, random prime-labeled, and used to screen an S. mansoni BAC genomic library [74] to isolate BAC clones that harbor the genomic sequence of receptor II. The isolated BAC clones were used as templates in PCR reactions to validate the information obtained from searching genomic DNA databases. The order of the assembled contigs was confirmed by PCR on genomic DNA extracted from S. mansoni cercariae as well as SmTβRII-positive BAC clones isolated from BAC library screening, using primers located on the 3′- and 5′-ends of each contig and the following one. In addition, the intron positions were confirmed by PCR amplification of DNA fragments that represent exon–intron boundaries or two adjacent exons, when such exons were interrupted by small introns.

### Production of polyclonal antibodies against SmTβRII.

The pSmTβRII-N(1-193)/TOPO-II was used to amplify a cDNA fragment encoding 153 aa from the N-terminal domain of the receptor and cloned into the prokaryotic expression vector, pMAL-c2x (New England Biolabs, Ipswich, Massachusetts, United States), downstream and in frame with the MBP (maltose-binding protein) coding sequence, pSmTβRII-N(41-193)/pMAL. A 67-kDa MBP fusion protein was expressed, purified following the manufacturer's instructions, and used to immunize a New Zealand White rabbit. A dose of 200 μg of the fusion protein emulsified in Freund's complete adjuvant, as a primary dose, was administered subcutaneously followed by 200 μg in Freund's incomplete adjuvant for two booster doses each at 4-wk intervals. An activating dose given intramuscularly (200 μg in 1× PBS) was used 7 d before sacrificing the animal. IgG fractions were affinity purified over a Protein A-Sepharose (Amersham Biosciences) and quantified. Pre-immune rabbit serum was processed similarly to provide reagents for negative controls. Affinity-purified IgG was used for immunoprecipitation, immunofluorescence, and Western blot analyses.

### Immunological assays.

Purified IgG fractions were used to detect the native protein in parasite extracts and in cryosections following protocols described in previous studies [37,38]. Immunofluorescence assays on whole-mount adult worms, either live or acetone fixed, were also conducted, following a protocol similar to that used for cryosections, to examine whether the native protein was exposed on the parasite surface. Briefly, adult worm pairs, either live or fixed for 5 min in ice-cold acetone, were incubated for 2 h at room temperature in MEM medium supplemented with 10% fetal bovine serum (Invitrogen), used as a blocking reagent. Incubation medium was replaced with test and control primary antibodies (IgG fractions), 5 μg/ml diluted in the above medium, and incubated overnight at 4 °C. Samples were washed with 1× PBS for four times, 5 min each, and then anti-primary, biotin-conjugated secondary antibodies (5 μg/ml; Molecular Probes. Invitrogen, Carlsbad, California, United States) were incubated for 1 h at room temperature followed by a washing step as before. Alexa Fluor-647–conjugated streptavidin (5 μg/ml; Molecular Probes, Invitrogen) was used to visualize the reactive antigen antibodies. Autofluorescence attributed to phenolic compounds could be visualized at yellow-green fluorescence wavelength (520 nm), but not at far-red fluorescence (680 nm). Cyrosections and whole-mount parasites were examined using a Bio-Rad MRC-1024 confocal microscope equipped with Krypton-Argon laser and 522-nm and 680-nm filters (Bio-Rad, Hercules, California, United States). Micrographs representing different laser sections of the samples were chosen to delineate the localization of specific and nonspecific fluorescence in the examined samples.

### Detection of SmTβRII transcripts: RT-PCR and FISH analyses.

Two approaches were employed to detect SmTβRII mRNA. A semi-quantitative analysis to estimate relative SmTβRII mRNA levels in different developmental stages was carried out by RT-PCR as previously described [38]. In addition, gene transcripts were localized by FISH. In the semi-quantitative RT-PCR assay, PCR products, amplified in separate reactions each using a specific primer pair, were separated by electrophoresis in 2% agarose gels, stained with ethidium bromide, analyzed using a gel-documentation system (GelDoc1000; Bio-Rad), and quantified using Quantity One software (version 4.2.3; Bio-Rad). Negative control PCR reactions using reverse transcription reaction mix lacking reverse transcriptase were also included. Specific primers for the *S. mansoni α-tubulin* gene (bp 424–444 and the complementary sequence of bp 777–801 as forward and reverse primers, respectively, yielding 378-bp PCR product) were used to amplify a PCR product that served as a constitutively transcribed control [75]. A specific primer pair for SmTβRII (forward primer, starts at bp 845; reverse primer, ends at bp 1,080), was used that yields a 236-bp PCR product. Primer pairs specific for *SmTβRI* (forward primer starts at bp 1,608, reverse primer ends at bp 1,894), and *SmGCP* (forward primer starts at bp 818, reverse primer ends at bp 1,114), were also used to amplify the corresponding fragments of these gene transcripts yielding products of 287 and 297 bp long, respectively. In addition to the above genes, *SmSmad1, SmSmad2,* and *SmSmad4* were similarly processed as control genes. The PCR primers and reaction conditions that were used to amplify those products were as previously described [38]. Due to differences in the quality of RNA preparations included in this assay, the volumes of input cDNA templates of each stage were varied according to normalization data using α-tubulin control. Also, due to differences in the abundance of the assayed cDNA species and in order to ensure that the amplification products were analyzed in the exponential phase and below saturation limits (PCR plateau), the number of PCR cycles used to amplify each cDNA was also varied. For α-tubulin, 24 cycles was used, whereas 27 cycles were used to amplify the PCR products for SmTβRII, SmSmad4, SmSmad2, and SmGCP, and 28 and 29 PCR cycles were used in the case of SmSmad1 and of SmTβRI, respectively. All variables were considered and compensated for in data analysis.

For the FISH assay, the SmTβRII RNA probes, a test probe and a negative control probe were prepared by in vitro transcribing and labeling of the anti-sense and sense strands of the N-terminal domain of SmTβRII (pSmTβRII-N (1-193)/TOPO-II) using Sp6 and T7 RNA polymerases (Ambion, Austin, Texas, United States), respectively, and digoxigenin-11-uridine-5′-triphosphate (DIG-11-UTP; Roche Applied Science, Indianapolis, Indiana, United States), following the manufacturers' suggested instructions. An RNA probe representing the anti-sense strand of the coding sequence of a schistosome eggshell protein, P14, was used as a positive control, which localizes the P14 transcripts only to female vitelline cells [76]. Probe hybridization and washing was performed using paraffin-embedded sections that were de-paraffinized, hydrated, hybridized, and washed according to the recommended instructions of the mRNA*locator*-Hyb Kit (Ambion). DIG-labeled probe detection was achieved by using an anti-DIG monoclonal antibody (0.25 μg /ml; Roche Applied Science), followed by a biotin-conjugated goat anti-mouse antibody (Molecular Probes, Invitrogen), and the antibody complexes were visualized using streptavidin-conjugated Alexa Fluor 647 (Molecular Probes, Invitrogen) in concentrations similar to that used in the immunofluorescence assays. Sections were examined by Bio-Rad MRC-1024 confocal microscope using 522-nm and 680-nm filters.

### SmTβRII SmTβRI in vitro interaction.

Full-length coding cDNA sequences of SmTβRII and SmTβRI were divided into three regions each, representing the main structural domains of both receptors. These regions were PCR-amplified from full-length cDNA clones and cloned into the BamHI and XhoI sites of the in vitro transcription/translation, S-protein tagged and non-tagged vectors; pCITE-4a and pCITE-2a, respectively (EMD Biosciences, Novagen, San Diego, California, United States). The three regions were the N-terminal regions, which represent the extracellular TM domains (SmTβRII, bp 65–645, 190 aa; and SmTβRI, bp 19–522, 170 aa), the kinase domains of SmTβRII (bp 845–1,773; 310 aa) and SmTβRI (bp 613–1,796; 395 aa), and the C-terminal regions that follow the kinase domains, SmTβRII (bp 1,709–2,520; 270 aa) and SmTβRI (bp 1,696–2,361; 221 aa). Structural domains were expressed in vitro using rabbit reticulocyte lysates (STP3; EMD Biosciences, Novagen), in which the S-tagged proteins were synthesized with unlabeled amino acid mix, while ^35^S-methionine was incorporated into the non-tagged peptides. The full-length receptors were also cloned in these vectors as described above. In vitro interaction reactions were assembled using 5 μl of the radiolabeled protein and 10 μl of each of the non-labeled proteins, and up to 5 nM of different human TGF-β ligands in 10 μl (TGF-β1, up to 1.0 nM; Activin A, 2.0 nM; and BMP2 and BMP4, 5.0 nM [R&D Systems, Minneapolis, Minnesota, United States]). Reaction volumes were brought up to 50 μl with 1× IPP buffer (150 mM NaCl, 20 mM Tris-HCl, [pH 7.5], 2% glycerol). The reactions were incubated for 1 h at room temperature after which protein complexes were precipitated by adding either 30 μl of 50% pre-washed S-protein beads (EMD Biosciences, Novagen), or 3 μg of a specific antibody reagent (IgG fraction) directed against one of the non-labeled proteins, which was then precipitated using Protein A-Sepharose beads (Amersham Biosciences). Reactions were rocked at room temperature for an extra 1 h, centrifuged, and then washed 4× with 1× IPP buffer containing 0.1% NP-40. Protein-bound beads were re-suspended in 1× SDS sample buffer, boiled, and separated by SDS-PAGE. The 12% gels were stained, destained, treated with the fluorographic reagent (Amplify; Amersham Biosciences), dried, and then exposed to X-ray film.

### Treatment of *S. mansoni* adult worms with human TGF-β1 and BMP2.

All culture media and supplements were purchased from Invitrogen, unless otherwise stated. Freshly perfused adult worms were collected from infected golden hamsters [77], washed, and then incubated overnight at 37 °C, 5% CO_2_ in MEM medium supplemented with 1 mM sodium pyruvate, 1× non-essential amino acid solution, 1× amino acid solution, 100 U/ml penicillin, 100 μg/ml streptomycin, 1 μg/ml amphotericin B (antibiotic antimycotic solution), and 2 mM Glutamax-I. Recombinant human TGF-β1 and BMP2 (R&D Systems) were added individually to about 25 pairs of adult worms to final concentrations of 1.0 nM and 5.0 nM, respectively, and incubation was continued for another 24 h, after which the medium was changed and worms were incubated for an additional 24 h. Total RNA was extracted from untreated or TGF-β–treated worm pairs, and cDNA was synthesized and then subjected to PCR using different specific gene primers following the optimized PCR conditions for each gene employing the aforementioned protocol. PCR products were separated onto 2% agarose gels, and processed and quantified as described above.

### Silencing of SmTβRII gene expression by siRNA treatment of S. mansoni worms.

Before conducting the RNAi experiments, conditions for schistosome transformation were optimized. The red fluorescent protein vector, pHcRed1-1 (BD Biosciences, Clontech, Palo Alto, California, United States) was used after modification by inserting the cytomegalovirus immediate early promoter *(P_CMV_)* upstream of the coding sequence yielding pCMV-HcRed. Nucleic acid (plasmids and RNA) delivery into schistosome worms was achieved by particle bombardment using the Helios Gene Gun particle delivery system (Bio-Rad). Twenty μg of vector (2-fold CsCl gradient purified) was used to prepare the bullets by coating 10 mg of 1-μm diameter gold microcarrier particles (2-μg DNA/mg gold particles) (Bio-Rad). A DNA/gold suspension was used to coat Teflon tubing (Tefzel; Bio-Rad) following the manufacturer's suggested conditions. Sample bullets were used to bombard 33-d-old worms, using different helium pressures. Each sample received two shots at the same pressure at 1 μg DNA/0.5 mg gold particles/shot. Transformed parasites were incubated in MEM medium containing 10% fetal bovine serum, 0.5% lactalbumin hydrolysate and streptomycin and gentamicin (100 μg/ml each), and penicillin (100 U./ml), at 37 °C, 5% CO_2_ for 24–48 h, and then evaluated using a Nikon FXA fluorescent microscope and a red filter (Nikon, Tokyo, Japan). For 33-d-old worms, 400 psi was determined to be the optimal helium pressure.

SmTβRII-specific RNAi was constructed by in vitro transcribing pSmTβRII-N (1–193)/TOPO-II from both directions using T7 and Sp6 bacteriophage RNA polymerases (MegaScript in vitro transcription kits; Ambion) in separate reactions. The DNA template was digested with DNaseI, and the resulting RNA strands were annealed, purified over a sizing column (ProbeQuant G-50 Micro Columns; Amersham Biosciences), spectrophotometrically quantified, and then an aliquot of about 12 μg was digested with 15 units of RNaseIII (Ambion) following the supplier's instructions. The resulting 20–30 bp dsRNA (double-stranded DNA) was further purified over a different type of sizing matrix (Micro Bio-Spin 6; Bio-Rad). An aliquot of each RNA sample, before and after digestion, was separated on a 2% agarose gel to assess the efficiency of the in vitro transcription process and the dsRNA digestion. siRNA was used to prepare the siRNA/gold bullets following the above protocol described for plasmid DNA except that the amount of dsRNA was 12 μg. Freshly perfused 26-d-old and 33-d-old worm pairs were used as target stages for particle bombardment using SmTβRII-N–siRNA bullets. The subsequent effect on SmTβRII and SmGCP gene expression were evaluated by RT-PCR analyses. Control parasites were bombarded using gold bullets. Worms were bombarded using 400-psi helium pressure. Transformed parasites were incubated for 24 h in the previously described serum-free culture medium, and either left untreated or treated for 24 h with TGF-β1 (1 nM). Two days after perfusion, the 28-d-old and 35-d-old parasites were processed for total RNA extraction, cDNA synthesis, and PCR amplification of α-tubulin, SmTβRII, SmTβRI, SmGCP, SmSmad1, SmSmad2, and SmSmad4. The PCR products were gel-separated, analyzed, and compared to the constitutively transcribed control, the α-tubulin, as described above.

## Supporting Information

Figure S1 TGF-β Receptor II Gene Showing the Relative Locations of the Primers and the Obtained PCR Products Used to Elucidate the 3′-End of *SmRK2* cDNA (A) A sketch represents part of TGF-β receptor II gene (exons 7 and 8, and partial sequence of 9) showing the relative locations of the primers used to elucidate the 3′-end of *SmRK2* cDNA. The pound sign (#) and asterisk (*) show the relative locations of the proposed poly A stretch in the Forrester et al. study [39] and the putative polyadenylation signal at the 3′-end of exon 8.
(B) Agarose gels showing PCR products amplified from S. mansoni adult worm pair cDNA templates (lanes 2), which were prepared from a total RNA that had been treated with DNaseI, genomic DNA extracted from S. mansoni adult worm pairs (lanes 3), and a SmTβRII-positive BAC clone (lanes 4). A minus RT control (lanes 1) was also included to verify absence of any genomic DNA contaminations in cDNA preparations. Forward primers used in these experiments are indicated to the left of each set of amplification reactions, and reverse primers are listed above lane numbers. Molecular size marker (M) was run, and sizes are indicated in kb to the right of each panel. Product sizes are listed in Table S2.
(1.6 MB TIF)

Figure S2 Whole-Mount Live S. mansoni Adult Worm Immunofluorescence (Negative Control) Live S. mansoni adult worms were probed with pre-immune rabbit IgG, followed by biotin-conjugated anti-rabbit IgG. Samples were incubated with streptavidin Alexa Fluor 647 conjugate and analyzed with Bio-Rad MRC1024 confocal laser microscope.
(A) represents the phase-contrast field of a male worm (♂) and a female worm (♀).
(B) represents autofluorescence field viewed at 522 nm.
(C) represents the far-red fluorescence field (680 nm). Please note, due to lack of specific fluorescence in panel (C) that results in the lack of visible details at this wavelength, the micrograph was set at high brightness to reveal the adult worms in the field.
O, esophagus; Os, oral sucker; V, vitellaria.
(2.2 MB TIF)

Table S1 Sequences of Primers Used in Mapping the 3′-End of SmRK2 cDNA and Their Locations Respective to Either cDNA or Genomic Gene (31 KB DOC)

Table S2 Expected Sizes of PCR Products of cDNA or Genomic DNA Resulted from Using Different Primer Combinations to Elucidate the Structure of the 3′-End of SmRK2 cDNA (28 KB DOC)

### Accession Numbers

Sequence data reported in this manuscript are available from GenBank (http://www.ncbi.nlm.nih.gov/Genbank) under accession number AY654287. Other GenBank accession numbers for the genes or genetic material discussed in this paper are as follows: *α-tubulin* (M80214); *SmGCP* (U47862); *SmRK2* (AY550912); *SmRK2a* (AY285784); and [deleted repeat of *SmRK2*] *SmTβRI* (AF031557).

## Abbreviations

aa: amino acids
BAC: bacterial artificial chromosome
bp: base pairs
ECD: extracellular domain
EST: expressed sequence tag
FISH: fluorescent in situ hybridization
kb: kilobase
RNAi: RNA interference
siRNA: short interfering RNA
SmGCP: Schistosoma mansoni gynaecophoral canal protein
SmTβRI: Schistosoma mansoni transforming growth factor-beta type I receptor
SmTβRII: Schistosoma mansoni transforming growth factor-beta type II receptor
TGF-β: transforming growth factor-beta
TM: transmembrane
