# Supplementary material for: Schistosoma mansoni TGF-β Receptor II: Role in Host Ligand-Induced Regulation of a Schistosome Target Gene
Source: PLoS Pathog. 2006 Jun 16;2(6):e54. doi: 10.1371/journal.ppat.0020054 (PMC1479047; doi:10.1371/journal.ppat.0020054)
Supplement: Supplementary file 1 [file ppat.0020054.st001.doc]

| Primer name | Length | Orientation | Sequence | Location | | |
| --- | --- | --- | --- | --- | --- | --- |
|  |  |  |  | SmRK2 cDNA | SmTβRII cDNA | Genomic gene |
| F1  R1  R2  F2  R3  R4  R5 | 22 mer  24 mer  22 mer  24 mer  24 mer  23 mer  22 mer | Fwd  Rev  Rev  Fwd  Rev  Rev  Rev | 5’-GGCGCTATTCAGTTTTCAAAGG-3’  5’-TGTTTGATTTGCAAAATAGTAGGG-3’  5’-CGTCACCAGAAGCGTGATATTC-3’  5’-TAGAATATCACGCTTCTGGTGACG-3’  5’-TAATCAACTTTGATTCAATCCGTC-3’  5’-CCCATACTAGGACAAAGCGACCG-3’  5’-GTAGTCAAACGTTTGGTTACAC-3’ | 1469-1490  ~1912-1935  N/A  N/A  N/A  N/A  N/A | 1433-1454  N/A  N/A  N/A  N/A  N/A  ~1752-1773 | 16,455-16,476  ~16,898-16,921  ~17,347-17,368  17,345-17,368  ~17,470-17,493  ~18,086-18,108  ~21,440-21,461 |
