# Supplementary material for: Schistosoma mansoni TGF-β Receptor II: Role in Host Ligand-Induced Regulation of a Schistosome Target Gene
Source: PLoS Pathog. 2006 Jun 16;2(6):e54. doi: 10.1371/journal.ppat.0020054 (PMC1479047; doi:10.1371/journal.ppat.0020054)
Supplement: Supplementary file 2 [file ppat.0020054.st002.doc]

|  | PCR Product Size (bp) | | | | | | | | | |
| --- | --- | --- | --- | --- | --- | --- | --- | --- | --- | --- |
| Reverse Primer | R1 | | R2 | | R3 | | R4 | | R5 | |
| Forward Primer | cDNA | Genomic | cDNA | Genomic | cDNA | Genomic | cDNA | Genomic | cDNA | Genomic |
| F1 | 467 | 467 | 914 | 914 | N/A | 1039 | N/A | 1654 | 341 | 5007 |
| F2 | N/A | N/A | N/A | N/A | N/A | 138 | N/A | 753 | N/A | 4117 |
